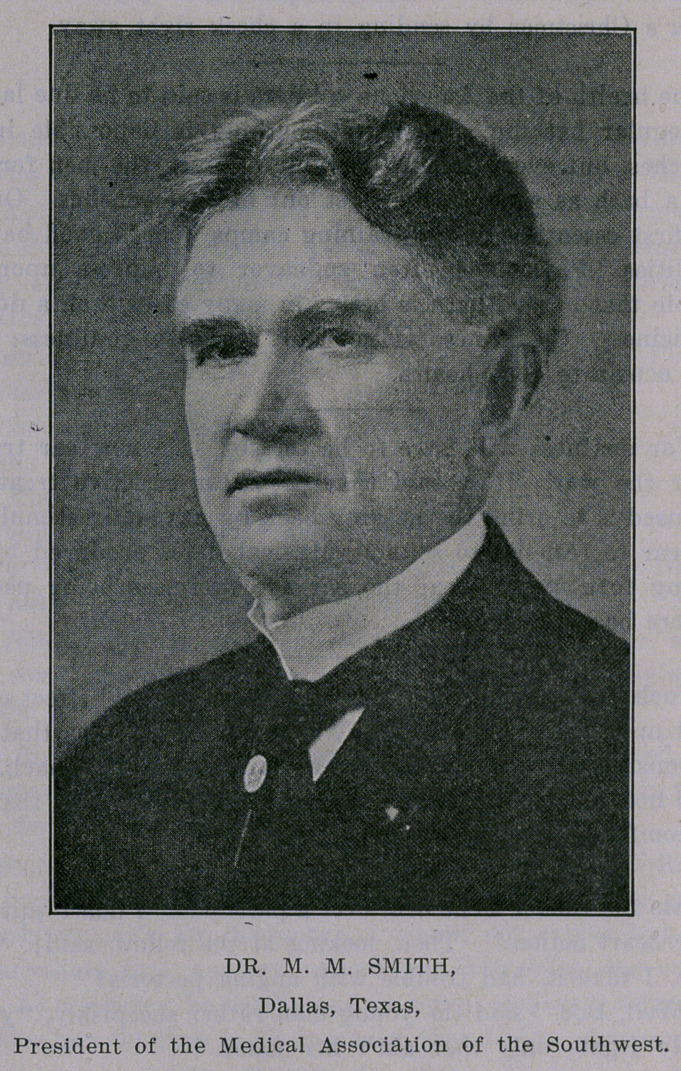# An Interesting Series of Articles

**Published:** 1918-12

**Authors:** 


### An Interesting Series of Articles.

The January, 1919, issue of this Journal will contain the first of a series of articles by Dr. Gustav Kolischer of Chicago. Ill.

Dr. Kolischer is the Professor of Genito-Urinary Surgery, Chicago Post Graduate School, Attending Surgeon to the Genito-Urinary and Radio-therapeutic Department of the Micheal Reese Hospital of Chicago.

The series consists of an article on Hypertrophy of the Prostate Gland;

The Technique of Suprapubic Prostatectomy;

Tumors of the Urinary Bladder;

The Pus Kidney;

Technique of Nephrotomy and Nephrectomy.

The articles have been prepared especially for this Journal by the author, who is an authority on these subjects.

The following letter from one who knows Dr. Kolischer, will convey some idea of the esteem in which he is held by those who know him personally:

Dear Mrs. Daniel: I have just read the article submitted by Dr. Gustav Kolischer, of Chicago, to you, for publication in the *Texas Medical Journal.*

I wish to thank you very much for allowing me the privilege of reading this splendid essay. After having had a close personal relation with Dr. Kolischer, I would not expect him to write anything that was not *exceedingly interesting and complete.* As a teacher, he hardly has an equal; and as a surgeon, I consider him the best in his specialty, that I have ever seen.

The five essays of Dr. Kolischer's will be almost a complete work on Gento-Urinary surgery, and I think anyone interested in that line of work can not afford to miss the opportunity to read these very valuable papers.

Very truly yours,

J. Gordon Bryson.

The publisher of this journal dislikes to remind its readers through its columns that subscription accounts should be settled, but three cents postage stamps run into dollars rapidly, and they do not always get the desired results. Suppose you, if you are a delinquent subscriber, try to put a little joy into the publisher's Christmas by sending in a check right away.

---

The health of the American soldiers is said to be due largely to regular bathing. Of course, bathing is impossible in the trenches, but every facility possible is given the men for taking a bath as soon as they get out of the trenches. One of the first essentials of the training camps, too, is good bathing facilities. Physicians often endeavor to impress upon the people that a good bath is better in many cases than a dose of medicine. Cleanliness is not only akin to godliness; it is first cousin to good health.

---

War deafness will have to be treated as a new ear trouble after the war. It is said that the most effective treatment yet used is to bring the hearing back by gradually stimulating the ear to respond to sound. Musical tones produced by the tuning fork struck near the ear are exercises being used to restore hearing.

---

Much has been said in times past about the evil effect of hot food upon the stomach. Dr. William L. Mayo says that it is responsible for a large part of the cancer of the stomach. He does not advise cold food, but urges the use of food that can be comfortably taken into the mouth.

---

SAID the Doctor as he bent over a patient: "I don't quite like your heart action." Then, looking at the fellow, said: "You have, I take it, had trouble with angina pectoris?"

"Well, Doc," said the young man rather sheepishly, "you're partly right; only that ain't her name."

---

THE LATE Dr. B——, of Bristol, who died very rich, coming into the bedroom of a patient a very few minutes after he had expired, perceived something glittering through the clenched fingers of one hand; he gently opened them, took out the guinea, and put it into his pocket, observing, "This was certainly intended for me!"

## Figures and Tables

**Figure f1:**